# Assessment of palpitations in patients with frequent premature ventricular contractions

**DOI:** 10.1111/jce.16476

**Published:** 2024-10-21

**Authors:** Yue Gao, Rui Jiang, Yan Liu, Zi‐xuan Li, Xin‐he Xu, Shi‐jie Li, Xian‐jin Li, Bing Han

**Affiliations:** ^1^ Graduate School of Bengbu Medical University Bengbu China; ^2^ Graduate School of the Clinical College of Xuzhou Medical University Xuzhou China; ^3^ Division of Cardiology Xuzhou Central Hospital Xuzhou China

**Keywords:** clinical characteristics, electrocardiographic characteristics, frequent premature ventricular contractions, palpitations, symptom‐rhythm correlations

## Abstract

**Introduction:**

In patients with frequent premature ventricular contractions (PVCs), palpitations may not always be directly caused by PVCs, and therefore, it is essential to establish symptom‐rhythm correlations to determine the appropriate treatment. This study aims to analyze the palpitations and related factors in patients with frequent PVCs.

**Methods:**

The study enrolled patients with frequent PVCs who were not combined with other arrhythmias or structural heart disease. Through face‐to‐face consultation, patients were divided into symptomatic and asymptomatic groups. For symptomatic patients, the correlation between palpitations and PVC was further evaluated based on the temporal consistency of symptom onset and PVC occurrence. The demographic, clinical, and electrocardiogram features of the patients in each group were compared.

**Results:**

Of the 214 patients enrolled, 124(57.9%) experienced palpitations. Compared to the asymptomatic group, the symptomatic group had a higher proportion of females (63.7% vs. 47.8%; *p* = .020) and a higher proportion of subjects with anxiety (44.4% vs.14.4%; *p* = .000). Within the symptomatic patients, 72 (33.60%) who had palpitations that were clearly correlated with PVCs were classified as the PVC‐relevant group. In this group, the PVC CI ratios were significantly lower (55% [52% −60%] vs. 62% [55% −67%]; *p* = .001) and the Post‐PVC CI were longer (1170 [1027–1270] vs. 1083 [960–1180] ms; *p* = .018) than in the PVC‐irrelevant group.

**Conclusion:**

A direct relationship between palpitations and PVCs could be established only in a minority of patients with frequent PVCs. PVCs with a relatively short PVC CI and a long post‐PVC CI were more likely to cause palpitations, whereas palpitations lasting only a few seconds were more likely to be directly relevant to PVCs.

## 1 INTRODUCTION

Premature ventricular contraction (PVC) is one of the most common arrhythmias. It has been detected in 40%–75% of apparently healthy individuals through 24–48 h Holter electrocardiogram (ECG) recording.^1^ In a study conducted in China, among patients who underwent 24‐h Holter monitoring due to palpitations, 67.7% of them had PVCs and 7.7% had frequent PVCs.^2^ Because the vast majority of PVC patients without noticeable cardiac structural abnormalities have a good prognosis,^3^ according to existing guidelines, the treatment of PVC is mainly based on the presence or absence of symptoms.^4^ The usual symptom of PVCs is palpitations, characterized by awareness of the heartbeat and described as unpleasant pulsations or sensations of movement in the chest and/or adjacent areas.^5^ There is a broad range of causes and underlying diseases associated with palpitations, including cardiac arrhythmia, structural heart diseases, psychosomatic disorders, and systemic diseases.^6^ Although arrhythmia is often the cause of palpitations, studies have shown only a weak relationship between palpitations and cardiac irregularities through ambulatory electrocardiographic monitoring.^5^ Some patients with PVC may experience palpitations, while others may be completely asymptomatic. On the other hand, for symptomatic patients, palpitations may not necessarily be directly relevant to PVCs themselves.^4^ It is essential to assess palpitations and establish symptom‐rhythm correlations to determine the appropriate treatment for PVCs. Further in‐depth research is needed in this area. This observational study aims to analyze the palpitations and related factors in patients with frequent PVCs.

## 2 METHODS

### 2.1 Study subjects

Patients with frequent PVCs who visited Xuzhou Central Hospital between September 2022 and May 2023 were included in this study. To be eligible for this study, participants had to meet the following criteria: (1) age between 18 and 80 years; (2) diagnosed with frequent PVCs (>1000 beats/24 h) based on 24‐h Holter recordings performed within the last month; (3) agreeing to join this study and signing an informed consent form. The exclusion criteria were as follows: (1) coexistence of other types of arrhythmia, including frequent premature atrial contractions, atrial tachycardia, atrial flutter, atrial fibrillation, atrioventricular reentrant tachycardia, nonpersistent or persistent ventricular tachycardia, sick sinus syndrome, or atrioventricular block; (2) existence of structural heart disease; (3) existence of heart failure (LVEF < 50%); (4) implantation of cardiac electronic devices; (5) history of cardiac surgery; (6) existence of serious other systemic diseases; (7) existence of severe mental disorders or language comprehension disorders; (8) incomplete clinical data. This study was approved by the Ethics Committee of Xuzhou Central Hospital. All patients have signed informed consent forms.

### 2.2 Demographic and clinical data collection

All patients' demographic characteristics and clinical data were collected, including marital status, education level, occupation, past history, drug use, tobacco and alcohol habits, and physical examination. In addition, the Zung's Self‐Rating Anxiety Scale was used to screen for anxiety with a cut‐off score of 40.^7^

### 2.3 12‐lead resting ECGs and Holter recordings analysis

In each patient, the standard 12‐lead resting ECG was recorded at a paper speed of 25 mm/sec and voltage gain of 10 mm/mV. The 12‐lead Holter ECG was recorded for 24 h. The ECG recordings were automatically analyzed by computers and manually checked by experienced cardiologists.

From the resting ECG, the following parameters were collected: (1) baseline sinus cycle length (SCL): the R‐R interval of the sinus beats just before the PVC; (2) PVC coupling interval (CI): from the onset of the R wave of the sinus beat immediately before the PVC to the onset of the R wave of the PVC; (3) PVC CI ratio (%): PVC CI/SCL × 100%; (4) Post‐PVC CI: from the onset of the R wave of the PVC to the onset of the R wave of the next sinus beat; (5) Post‐PVC CI ratio (%): post‐PVC CI/SCL × 100%; (6) PVC QRS width: from the onset of the PVC QRS complex to the end of the S wave; (7) PVC origin: based on the morphology of QRS complex, PVCs were distinguished into outflow tract origin (with positive QRS in inferior leads) and non‐outflow tract origin (with negative QRS in inferior leads). For multiple PVCs, we chose to measure ECG parameters with PVCs that predominated in the Holter recordings and were traced in the resting ECG.

From the Holter ECG, the following parameters were collected: (1) PVC burden: the percentage of the total number of PVCs to the total number of heartbeats during Holter monitoring; (2)circadian distribution of PVCs: according to the circadian distribution characteristics of PVCs, they were distinguished into daytime‐dominant pattern (the proportion of PVCs during daytime exceeded 70%), nighttime‐dominant pattern (the proportion of PVCs during nighttime exceeded 70%) and uniform‐distribution pattern (the difference in the proportion of PVC during daytime and nighttime day did not exceed 40%). Daytime was defined as the period from 6 am to 6 pm and night as the period from 6 p.m. to 6 a.m. the following day; (3) correlation between PVC frequency and heart rate (HR): the relationship between the number of PVCs per hour and the hourly mean HR was analyzed by Pearson's correlation and presented as positive, negative or no correlation.

### 2.4 Symptom assessment and patient grouping

Whether patients had the symptoms of palpitations within the past month was confirmed through face‐to‐face consultation. Based on the presence or absence of palpitations, patients were divided into symptomatic and asymptomatic groups. Among the symptomatic patients, those with a temporal consistency between the occurrence of palpitations and the appearance of PVCs were classified as PVC‐relevant, while those without this clear correlation were classified as PVC‐irrelevant. To clarify the relevance of palpitations to PVCs, we identified the occurrence of PVCs through in‐office ECG recording or physical examination and asked patients whether they experienced palpitations and felt the occurrence of PVCs at the same time. The clinical features associated with palpitations were collected, including the chronological order of symptom onset and PVC diagnosis, the duration of palpitations, and the correlation between palpitations and physical activity. The severity of palpitations was evaluated using the Visual Analog Scale (VAS).^8^ Patients' demographic, clinical and ECG characteristics were compared between these groups.

### 2.5 Statistical analysis

Normally distributed continuous variables were expressed as mean ± standard deviation (SD) and compared by the Student *t*‐test; non‐normally distributed data were presented as the median with interquartile range (IQR) and compared by the Mann‐Whitney U test. Categorical variables were described in numbers or percentages and compared through the χ2 test. Pearson's correlation was performed to analyze the linear relationship between continuous variables. A two‐sided *p* < .05 was considered statistically significant. Statistical analysis of the data was performed with SPSS 26.0 software.

## 3 RESULTS

### 3.1 Demographic and clinical characteristics

A total of 214 patients were enrolled in this study, with 92 (43.0%) being male and the mean age being mean age was 56.6 ± 17.2 years. The demographic and clinical characteristics are shown in Table 1.

**Table 1:** Baseline demographics and clinical characteristics.

Characteristic	Total (*n* = 214)
**Demographic characteristics**	
Females [*n* (%)]	122 (57.0)
Age (years) (X ± S)	56.6 ± 17.2
Age group	
≤20 (years) [*n* (%)]	12 (5.6)
21–65 (years) [*n* (%)]	125 (58.4)
>65 (years) [*n* (%)]	77 (36.0)
Marital status	
Single [*n* (%)]	20 (9.3)
Married [*n* (%)]	185 (86.4)
Divorced/widowed [*n* (%)]	9 (4.3)
Education level	
Primary school and below [*n* (%)]	73 (34.1)
Junior and senior high school [*n* (%)]	80 (37.4)
University and above [*n* (%)]	61 (28.5)
Type of occupation	
Intellectual worker [*n* (%)]	52 (24.3)
Physical worker [*n* (%)]	65 (30.4)
Unemployed [*n* (%)]	49 (22.9)
Retirees [*n* (%)]	48 (22.4)
BMI (kg/m^2^) (X ± S)	24.8 ± 3.5
**Clinical characteristics**	
PVC diagnostics	
Detected during medical exams [*n* (%)]	141 (65.9)
Consultation for palpitations [*n* (%)]	73 (34.1)
Course of PVC (month) [M(Q)]	20 (15–50)
Current smoking [*n* (%)]	64 (29.9)
Current drinking [*n* (%)]	63 (29.4)
Comorbidity	
Coronary artery disease [*n* (%)]	50 (23.4)
Hypertension [*n* (%)]	78 (36.4)
Stroke [*n* (%)]	23 (10.7)
Diabetes [*n* (%)]	33 (15.4)
Use of medicines	
Antiarrhythmic drugs [*n* (%)]	44 (20.6)
β‐blocker [*n* (%)]	63 (29.4)
Anxiety based on SAS [*n* (%)]	68 (31.7)

Abbreviations: BMI, body mass index; PVC, premature ventricular contraction; SAS, Self‐Rating Anxiety Scale.

### 3.2 ECG characteristics related to PVCs

In these patients, the mean total number of PVCs was 12828 beats/24 h (IQR, 5986–22219), and the mean PVC burden was 13.0% (IQR, 5.9%–21.3%). ECG characteristics of PVCs are displayed in Table 2.

**Table 2:** Electrocardiographic characteristics of all patients.

Characteristics	Total (*n* = 214)
**Resting ECG**	
Sinus cycle length (ms) [M(Q)]	800 (720–860)
PVC CI (ms) [M(Q)]	454 (414–511)
PVC CI ratio (%) [M(Q)]	58 (53–64)
Post‐PVC CI (ms) [M(Q)]	1116 (1000–1213)
Post‐PVC CI ratio (%) [M(Q)]	141 (134–147)
PVC QRS width (ms) [M(Q)]	140 (130–150)
PVC Origin	
Outflow tract [*n* (%)]	159 (74.3)
Non‐outflow tract [*n* (%)]	55 (25.7)
**Holter ECG**	
Total number of PVCs [M(Q)]	12828 (5986–22219)
PVC burden (%) [M(Q)]	13.0 (5.9‐21.3)
Circadian distribution of PVCs	
Daytime‐dominant [*n* (%)]	46 (21.5)
Nighttime‐dominant [*n* (%)]	21 (9.8)
Uniform‐distribution [*n* (%)]	147 (68.7)
PVC frequency ‐ heart rate correlation	
Positive [*n* (%)]	101 (47.2)
Negative [*n* (%)]	23 (10.7)
No correlation [*n* (%)]	90 (42.1)

Abbreviations: CI, coupling interval; ECG, electrocardiography; PVCs, premature ventricular contractions.

### 3.3 Findings of palpitations assessment and comparative analysis between groups

**Figure 1:**
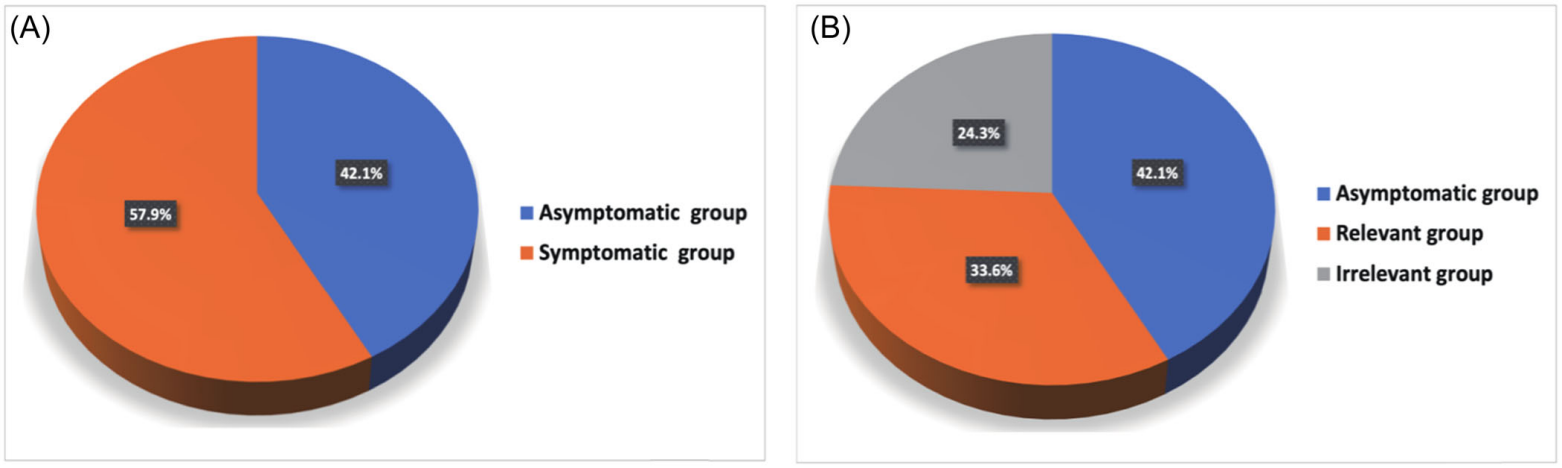
Pie charts showing the proportions of patients with and without palpitations (A) and the proportion of those with PVC‐relevant palpitations (B). PVC, premature ventricular contraction.

According to the face‐to‐face consultations,124 out of 214 patients (57.9%) experienced palpitation symptoms within the last month, while the remaining 90 patients (42.1%) did not report any palpitations (Figure 1). Compared to the asymptomatic group, the symptomatic group had a higher proportion of females (63.7% vs. 47.8%; *p* = .020), a lower proportion of subjects aged 20 years or younger, a higher proportion of subjects aged 21–65 years (≤20 years: 2.4% vs. 10.0%; 21–65 years: 64.5% vs. 50.0%; >65 years: 33.1% vs. 40.0%; *p* = .019), a higher proportion of subjects takingβ‐blocker (37.9% vs. 17.8%; *p* = .001), a higher proportion of subjects with anxiety (44.4% vs. 14.4%; *p* = .000), and longer disease courses (28 [16–74] vs. 17 [12–38] months; *p* = .002). The comparisons between the symptomatic and asymptomatic groups are summarized in Table 3.

**Table 3:** Comparison between the symptomatic and asymptomatic groups.

**Characteristics**	**Asymptomatic (*n*** = **90)**	**Symptomatic (*n*** = **124)**	***p* value**
**Demographic characteristics**			
Females [*n* (%)]	43 (47.8)	79 (63.7)	.020
Age (years) (X ± S)	55.5 ± 20.5	57.5 ± 14.4	.427
Age group			.019
≤20 (years) [*n* (%)]	9 (10.0)	3 (2.4)	
21–65 (years) [*n* (%)]	45 (50.0)	80 (64.5)	
>65 (years) [n (%)]	36 (40.0)	41 (33.1)	
Marital status			.017
Single [*n* (%)]	14 (15.6)	6 (4.8)	
Married [*n* (%)]	74 (82.2)	111 (89.6)	
Divorced/widowed [*n* (%)]	2 (2.2)	7 (5.6)	
Education level			.868
Primary school and below [*n* (%)]	31 (34.4)	42 (33.9)	
Junior and senior high school [*n* (%)]	35 (38.9)	45 (36.3)	
University and above [*n* (%)]	24 (26.7)	37 (29.8)	
Type of occupation			.277
Intellectual worker [*n* (%)]	25 (27.8)	27 (21.8)	
Physical worker [*n* (%)]	30 (33.3)	35 (28.2)	
Unemployed [*n* (%)]	15 (16.7)	34 (27.4)	
Retirees [*n* (%)]	20 (22.2)	28 (22.6)	
BMI (kg/m^2^) (X ± S)	24.5 ± 3.5	25.1 ± 3.4	.181
**Clinical characteristics**			
Course of PVC (Month) [M(Q)]	17 (12–38)	28 (16–74)	.002
Current smoking [n (%)]	32 (35.6)	32 (25.8)	.124
Current drinking [n (%)]	29 (32.2)	34 (27.4)	.447
Comorbidity			
Coronary artery disease [*n* (%)]	24 (26.7)	26 (21.0)	.331
Hypertension [*n* (%)]	32 (35.6)	46 (37.1)	.817
Stroke [*n* (%)]	11 (12.2)	12 (9.7)	.553
Diabetes [*n* (%)]	15 (16.7)	18 (14.5)	.667
Use of medicines			
Antiarrhythmic drugs [*n* (%)]	13 (14.4)	31 (25.0)	.059
β‐blocker [*n* (%)]	16 (17.8)	47 (37.9)	.001
Anxiety based on SAS [*n* (%)]	13 (14.4)	55 (44.4)	.000
**Electrocardiogram characteristics**			
**Resting ECG**			
Sinus cycle length(ms) [M(Q)]	800 (720–850)	800 (721–865)	.958
PVC CI (ms) [M(Q)]	458 (408–518)	450 (414–508)	.566
PVC CI ratio (%) [M(Q)]	59 (53–64)	57 (53–64)	.489
Post‐PVC CI (ms) [M(Q)]	1080 (982–1200)	1120 (1000–1240)	.139
Post‐PVC CI ratio (%) [M(Q)]	140 (133–145)	142 (134–148)	.107
PVC QRS width (ms) [M(Q)]	140 (130–150)	140 (130–160)	.420
PVC Origin			.220
Outflow tract [*n* (%)]	63 (70.0)	96 (77.4)	
Non‐outflow tract [*n* (%)]	27 (30.0)	28 (22.6)	
**Holter ECG**			
Total number of PVCs [M(Q)]	14765 (5515–22773)	11875 (6119–21226)	.474
PVC burden (%) [M(Q)]	14.8 (5.3–21.6)	11.4 (6.0–21.2)	.512
Circadian distribution of PVCs			.820
Daytime‐dominant [*n* (%)]	20 (22.2)	26 (21.0)	
Nighttime‐dominant [*n* (%)]	10 (11.1)	11 (8.9)	
Uniform‐distribution [*n* (%)]	60 (66.7)	87 (70.1)	
PVC frequency ‐ heart rate correlation			.124
Positive [*n* (%)]	38 (42.2)	63 (50.8)	
Negative [*n* (%)]	14 (15.6)	9 (7.3)	
No correlation [*n* (%)]	38 (42.2)	52 (41.9)	

Abbreviations: BMI, body mass index; CI, coupling interval; ECG, electrocardiography; PVC, premature ventricular contraction; SAS, Self‐Rating Anxiety Scale.

Out of the symptomatic patients, 72 (33.60%) with palpitations that had been demonstrated to be clearly correlated with PVCs were classified as the PVC‐relevant group and the other 52 (24.30%) with palpitations that were not definitely related to PVCs as the PVC‐irrelevant group (Figure 1). The only difference in the clinical characteristics between the two groups was the duration of the palpitation. In the PVC‐relevant group, more patients experienced palpitation that lasted only a few seconds (29.2% vs. 11.5%; *p*= .027). In addition, a higher proportion of patients took antiarrhythmic drugs (33.3% vs. 13.5%; *p* = .012). A comparison of ECG features between the two groups revealed significantly lower PVC CI ratios (55% [52% −60%] vs. 62% [55% −67%]; *p* = .001) and longer Post‐PVC CI (1170 [1027‐ 1270] vs. 1083 [960–1180] ms; *p* = .018) in the PVC‐relevant group. The comparisons between the PVC‐relevant and PVC‐irrelevant groups are presented in Table 4.

**Table 4:** Comparison between the PVC‐relevant and PVC‐irrelevant groups.

Characteristics	Relevant (*n* = 72)	Irrelevant (*n* = 52)	*p* value
**Demographic characteristics**			
Females [*n* (%)]	45 (62.5)	34 (65.4)	.742
Age (years) (X ± S)	58.3 ± 13.3	56.3 ± 15.8	.597
Age group			.635
≤20 (years) [*n* (%)]	1 (1.4)	2 (3.8)	
21–65 (years) [*n* (%)]	46 (63.9)	34 (65.4)	
>65 (years) [*n* (%)]	25 (34.7)	16 (30.8)	
Marital status			.917
Single [*n* (%)]	3 (4.2)	3 (5.8)	
Married [*n* (%)]	65 (90.3)	46 (88.4)	
Divorced/widowed [*n* (%)]	4 (5.5)	3 (5.8)	
Education level			.188
Primary school and below [*n* (%)]	23 (31.9)	19 (36.5)	
Junior and senior high school [*n* (%)]	23 (31.9)	22 (42.3)	
University and above [*n* (%)]	26 (36.2)	11 (21.2)	
Type of occupation			.110
Intellectual worker [*n* (%)]	16 (22.2)	11 (21.2)	
Physical worker [*n* (%)]	20 (27.8)	15 (28.8)	
Unemployed [*n* (%)]	15 (20.8)	19 (36.5)	
Retirees [*n* (%)]	21 (29.2)	7 (13.5)	
BMI (kg/m^2^) (X ± S)	25.0 ± 3.4	25.1 ± 3.4	.886
**Clinical characteristics**			
PVC diagnostics			.334
Detected during medical exams [*n* (%)]	27 (37.5)	24 (46.2)	
Consultation for palpitations [*n* (%)]	45 (62.5)	28 (53.8)	
Course of PVC (month) [M(Q)	37 (16–95)	21 (15–50)	.123
Characteristics of palpitations			
Severity based on VAS [M(Q)]	6 (4–8)	5 (4–7)	.311
The duration of the palpitation			.027
Within seconds [*n* (%)]	21 (29.2)	6 (11.5)	
Longer duration [*n* (%)]	51 (70.8)	46 (88.5)	
Symptoms and physical activity Correlation			.658
Positive [*n* (%)]	28 (38.9)	24 (46.1)	
Negative [*n* (%)]	8 (11.1)	4 (7.7)	
No correlation [*n* (%)]	36 (50.0)	24 (46.2)	
Current smoking [*n* (%)]	21 (29.2)	11 (21.2)	.314
Current drinking [*n* (%)]	21 (29.2)	13 (25.0)	.608
Comorbidity			
Coronary artery disease [*n* (%)]	16 (22.2)	10 (19.2)	.686
Hypertension [*n* (%)]	26 (36.1)	20 (38.5)	0.789
Stroke [*n* (%)]	8 (11.1)	4 (7.7)	0.525
Diabetes [*n* (%)]	9 (12.5)	9 (17.3)	0.453
Use of medicines			
Antiarrhythmic drugs [*n* (%)]	24 (33.3)	7 (13.5)	0.012
β‐blocker [*n* (%)]	27 (37.5)	20 (38.5)	0.913
Anxiety based on SAS [*n* (%)]	33 (45.8)	22 (42.3)	0.697
**Electrocardiogram characteristics**			
**Resting ECG**			
Sinus cycle length(ms) [M(Q)]	809 (732–873)	785 (690–844)	0.058
PVC CI (ms) [M(Q)]	452 (400–500)	448 (420–514)	0.290
PVC CI ratio (%) [M(Q)]	55 (52–60)	62 (55–67)	0.001
Post‐PVC CI (ms) [M(Q)]	1170 (1027–1270)	1083 (960–1180)	0.018
Post‐PVC CI ratio (%) [M(Q)]	144 (136–149)	140 (132–145)	0.056
PVC QRS width (ms) [M(Q)]	140 (122–160)	140 (130–158)	0.664
PVC Origin			0.911
Outflow tract [*n* (%)]	56 (77.8)	40 (76.9)	
Nonoutflow tract [*n* (%)]	16 (22.2)	12 (23.1)	
**Holter ECG**			
Total number of PVCs [M(Q)]	12828 (6606–21162)	10396 (5104–21461)	0.292
PVC burden (%) [M(Q)]	13.1 (6.8–21.2)	9.6 (5.1–21.3)	0.270
Circadian distribution of PVCs			0.651
Daytime‐dominant [*n* (%)]	16 (22.2)	10 (19.2)	
Nighttime‐dominant [*n* (%)]	5 (6.8)	6 (11.6)	
Uniform‐distribution [*n* (%)]	51 (70.8)	36 (69.2)	
PVC frequency ‐ heart rate correlation			0.522
Positive [*n* (%)]	35 (48.6)	28 (53.9)	
Negative [*n* (%)]	4 (5.6)	5 (9.6)	
No correlation [*n* (%)]	33 (45.8)	19 (36.5)	

Abbreviations: BMI, body mass index; CI, coupling interval; ECG, electrocardiography; PVC, premature ventricular contraction; SAS, Self‐Rating Anxiety Scale; VAS, Visual Analog Scale.

## 4 DISCUSSION

The main findings of this study included: (1) Almost half of patients with frequent PVCs were asymptomatic, and only one‐third had palpitations clearly correlated with PVCs; (2) Females were more likely to experience palpitations; (3) Young people had a lower likelihood of experiencing symptoms compared to adults; (4) Patients with palpitations had a higher chance of developing anxiety; (5) Patients with a lower PVC CI ratio and longer Post PVC CI were more likely to have PVC‐relevant palpitations.

In this study, 58% of patients with frequent PVCs complained of palpitations, which is comparable to previous studies.^9^, ^10^ Compared to asymptomatic patients, females make up a higher proportion of those with palpitations. A study by Vicent et al. revealed that palpitations were the most common reason for consultation in females.^11^ When comparing the palpitation‐PVC‐relevant group and the PVC‐irrelevant group, we found no gender difference. This suggests that there is no significant difference in the feeling of PVCs between males and females, while females might experience palpitations more often due to causes other than PVCs. According to Ehlers et al., females were more likely than males to be aware of sinus rhythm,^12^ and sinus tachycardia is also a common cause of palpitations.

In the comparison of symptomatic and asymptomatic patients, although there was no difference on average age, the proportion of symptomatic patients was significantly lower in those under 20 years of age, with only 3 out of 12 (25%) having palpitations. This is generally consistent with the results of previous studies.^13^ On the other hand, in the 21–65 age group, the proportion of symptomatic patients was obviously higher. In this age group, no significant difference in proportion was found when comparing patients in the PVC‐relevant group with those in the PVC‐irrelevant group, indicating that palpitations were more likely to be caused by reasons other than PVC.

Liang et al. discovered that 27% of patients with PVCs had depression,^14^ which is similar to the 32% in our group. In this study, anxiety was significantly more prevalent in those with palpitations than in those without. There may be a reciprocal causal relationship between palpitations and depression. Barsky et al. found that 45% of those with palpitations had anxiety or depressive disorder.^15^ They also observed that the correlation between palpitations and arrhythmia was weaker in patients with more severe psychiatric symptoms,^16^ suggesting that patients with depression and anxiety are more likely to have palpitations unrelated to arrhythmia.

Palpitations experienced by patients with PVCs are not necessarily directly related to PVCs and may be caused by other arrhythmias or non‐arrhythmic factors. In the present study, we excluded patients with coexisting other arrhythmias, but the role of sinus tachycardia, a common cause of palpitations, could not be ruled out. To establish a definite correlation between palpitations and PVCs, detailed face‐to‐face counseling was conducted in each of our patients, and a reliable judgment was made based on the temporal consistency of symptom onset and the detection of PVCs on ECG recording or physical examination. In this study, a clear correlation between palpitations and PVCs could be established in only 34%, suggesting that further confirmation of the correlation between symptoms and arrhythmia is necessary to determine treatment options for patients with PVCs. In contrast to patients in the PVC‐irrelevant group, a higher proportion of those in the PVC‐relevant group experienced palpitations lasting only a few seconds, indicating that short‐duration palpitations are more likely to be directly caused by PVCs.

The present study also revealed that patients with a relatively low PVC CI ratio and long Post‐PVC CI were more likely to experience PVC‐relevant palpitations. Previous studies have presented conflicting results regarding the role of PVC CI. Some studies have indicated that adverse left ventricular (LV) remodeling and dyssynchrony are more pronounced in patients with longer PVC CI,^17^, ^18^ whereas others suggested that shorter CI is associated with LV systolic dysfunction and reduced cardiac output.^19^, ^20^ Previous studies have also shown inconsistent findings on the impact of PVC CI on the symptoms of palpitations. According to Park et al., the PVC CI ratio was higher in individuals with symptoms than those without.^21^ In contrast, Takahashi et al. found that reduced PVC E wave flow was independently linked to PVC‐related syndromes, and E wave flow decreased with the shortening of CI.^22^ In their results, compared with asymptomatic patients, symptomatic patients had slightly shorter PVC CI and lower CI ratio. PVC‐relevant palpitations may occur due to an enhanced heartbeat resulting from prolonged ventricular filling time after PVC, known as postextrasystolic potentiation.^4^ Animal experiments have demonstrated that shortening the PVC CI will lead to stronger postextrasystolic potentiation,^23^ which may explain why PVCs with a shorter CI are more likely to cause palpitations.

### 4.1 Limitations of the study

This was a single‐center study with a limited sample size. The correlation between PVCs and palpitations was judged based on the temporal consistency between the onset of symptoms and the detection of arrhythmias on ECG recordings or physical examination, which might be challenging for patients with infrequent symptoms or a small number of PVCs. In addition to palpitations, PVC patients may experience other atypical symptoms, which may affect the judgment of the presence or absence of palpitations and their relevance to PVC. Moreover, approximately 30% of patients in this study were taking β‐blockers, with a higher proportion in the symptomatic group. Previous studies have indicated that β‐blockers might alleviate palpitations in PCV patients due to placebo effects.^24^ Considering these factors, the possibility of misjudgments could not be ruled out completely. In addition, because the ECG features of PVCs may have a certain degree of variability, the analysis based solely on one Holter or resting ECG recordings may also introduce bias and impact the analysis results.

## 5 CONCLUSION

A direct relationship between palpitations and PVCs could be established only in a minority of patients with frequent PVCs. Therefore, when making treatment decisions, it's important to carefully investigate the correlation between symptoms and arrhythmias. PVCs with a relatively short PVC CI and a long post‐PVC CI were more likely to cause palpitations, whereas palpitations lasting only a few seconds were more likely to be directly relevant to PVCs.

## Data Availability

The data that support the findings of this study are available on request from the corresponding author. The data are not publicly available due to privacy or ethical restrictions.

## References

[1] Ng GA . Treating patients with ventricular ectopic beats. Heart. 2006;92:1707‐1712. 17041126 10.1136/hrt.2005.067843 PMC1861260

[2] Dong Y , Li X , Zheng W , et al. Prevalence and heart rate variability characteristics of premature ventricular contractions detected by 24‐hour Holter among outpatients with palpitations in China: a cross‐sectional study. BMJ Open. 2022;12:e059337. 10.1136/bmjopen-2021-059337 PMC9351320 35918118

[3] Scorza R , Jonsson M , Friberg L , Rosenqvist M , Frykman V . Prognostic implication of premature ventricular contractions in patients without structural heart disease. EP Europace. 2023;25:517‐525. 10.1093/europace/euac184 PMC9935042 36261245

[4] Marcus GM . Evaluation and management of premature ventricular complexes. Circulation. 2020;141:1404‐1418. 32339046 10.1161/CIRCULATIONAHA.119.042434

[5] Barsky AJ . Palpitations, arrhythmias, and awareness of cardiac activity. Ann Intern Med. 2001;134:832‐837. 11346318 10.7326/0003-4819-134-9_part_2-200105011-00006

[6] Raviele A , Giada F , Bergfeldt L , et al. Management of patients with palpitations: a position paper from the European Heart Rhythm Association. Europace. 2011;13:920‐934. 21697315 10.1093/europace/eur130

[7] Dunstan DA , Scott N . Norms for Zung's Self‐rating Anxiety Scale. BMC Psychiatry. 2020;20:90. 32111187 10.1186/s12888-019-2427-6 PMC7048044

[8] Channer KS , Papouchado M , James MA , Pitcher DW , Rees JR . Towards improved control of atrial fibrillation. Eur Heart J. 1987;8:141‐147. 10.1093/oxfordjournals.eurheartj.a062241 3552679

[9] Hwang JK , Park SJ , On YK , Kim JS , Park KM . Clinical characteristics and features of frequent idiopathic ventricular premature complexes in the Korean population. Korean Circ J. 2015;45:391‐397. 26413107 10.4070/kcj.2015.45.5.391 PMC4580698

[10] Sukru A , Ozan AH , Furkan DM , et al. Effects of premature ventricular complex burden on left ventricular global longitudinal strain in patients without structural heart disease. J Clin Med. 2024;13:1796. 38542020 10.3390/jcm13061796 PMC10971011

[11] Vicent L , Rosillo N , Moreno G , et al. Sex differences in patterns of referral and resource utilization in the cardiology clinic: an outpatient analysis. Front Cardiovasc Med. 2023;10:1202960. 37588036 10.3389/fcvm.2023.1202960 PMC10425536

[12] Ehlers A , Mayou RA , Sprigings DC , Birkhead J . Psychological and perceptual factors associated with arrhythmias and benign palpitations. Psychosom Med. 2000;62:693‐702. 11020100 10.1097/00006842-200009000-00014

[13] Porcedda G , Brambilla A , Favilli S , Spaziani G , Mascia G , Giaccardi M . Frequent ventricular premature beats in children and adolescents: natural history and relationship with sport activity in a long‐term follow‐up. Pediatr Cardiol. 2020;41:123‐128. 31712859 10.1007/s00246-019-02233-w

[14] Liang J , Huang C , Yang B , et al. Depressive symptoms and risk factors in Chinese patients with premature ventricular contractions without structural heart disease. Clin Cardiol. 2009;32:E11‐E17. 10.1002/clc.20460 PMC6653526 19816869

[15] Barsky AJ , Cleary PD , Coeytaux RR , Ruskin JN . Psychiatric disorders in medical outpatients complaining of palpitations. J Gen Intern Med. 1994;9:306‐313. 8077994 10.1007/BF02599176

[16] Barsky AJ , Cleary PD , Barnett MC , Christiansen CL , Ruskin JN . The accuracy of symptom reporting by patients complaining of palpitations. Am J Med. 1994;97:214‐221. 8092169 10.1016/0002-9343(94)90003-5

[17] Voskoboinik A , Hadjis A , Alhede C , et al. Predictors of adverse outcome in patients with frequent premature ventricular complexes: the ABC‐VT risk score. Heart Rhythm. 2020;17:1066‐1074. 32109563 10.1016/j.hrthm.2020.02.020

[18] Potfay J , Kaszala K , Tan AY , et al. Abnormal left ventricular mechanics of ventricular ectopic beats: insights into origin and coupling interval in premature ventricular Contraction‐Induced cardiomyopathy. Circ Arrhythm Electrophysiol. 2015;8:1194‐1200. 26297787 10.1161/CIRCEP.115.003047 PMC4618025

[19] Sun Y , Blom NA , Yu Y , et al. The influence of premature ventricular contractions on left ventricular function in asymptomatic children without structural heart disease: an echocardiographic evaluation. Int J Cardiac Imaging. 2003;19:295‐299. 10.1023/a:1025418531853 14598897

[20] Abadir S , Blanchet C , Fournier A , et al. Characteristics of premature ventricular contractions in healthy children and their impact on left ventricular function. Heart Rhythm. 2016;13:2144‐2148. 27392943 10.1016/j.hrthm.2016.07.002

[21] Park KM , Im SI , Chun KJ , et al. Coupling interval ratio is associated with ventricular premature complex‐related symptoms. Korean Circ J. 2015;45:294‐300. 26240583 10.4070/kcj.2015.45.4.294 PMC4521107

[22] Takahashi S , Mine T , Ashida K , Kishima H , Masuyama T , Ishihara M . Left ventricular inflow velocity pattern in patients with symptomatic premature ventricular contraction. Circ J. 2019;84:26‐32. 31801920 10.1253/circj.CJ-19-0605

[23] Cooper MW , Lutherer LO , Lust RM . Postextrasystolic potentiation and echocardiography: the effect of varying basic heart rate, extrasystolic coupling interval and postextrasystolic interval. Circulation. 1982;66:771‐776. 6180844 10.1161/01.cir.66.4.771

[24] Krittayaphong R , Bhuripanyo K , Punlee K , Kangkagate C , Chaithiraphan S . Effect of atenolol on symptomatic ventricular arrhythmia without structural heart disease: a randomized placebo‐controlled study. Am Heart J. 2002;144:e10. 10.1067/mhj.2002.125516 12486439

